# Paternal consent in prenatal research: ethical aspects

**DOI:** 10.1007/s11019-019-09919-1

**Published:** 2019-08-10

**Authors:** Mats Johansson, Göran Hermerén, Nils-Eric Sahlin

**Affiliations:** Department of Clinical Sciences, Lund, Lund University, BMCI12, 221 84 Lund, Sweden

**Keywords:** Prenatal research, Informed consent, Paternal consent, Prenatal therapy

## Abstract

The role of mothers in prenatal research has been discussed extensively. Significantly less work has been done on the father’s role. In this article, focusing on ethical issues, we seek to redress this imbalance. Examining the father’s position in research conducted on pregnant women, we ask whether or not paternal consent ought to be required in addition to that of the pregnant woman. Having distinguished between different concepts of father and mother, we proceed by giving an overview of the reasons for requiring consent of the woman who is carrying the child. We then examine which of these reasons apply to the biological father, and show that some of them are relevant to the father. The case, roughly speaking, revolves around privacy issues, the father’s future legal responsibilities, and the likelihood that he will care about the health and wellbeing of his future child. These factors in the decision problem should all be recognized, as should the fact that they can in principle be trumped by other considerations.

## Introduction

The principle of informed consent is a cornerstone of contemporary research ethics. Human research subjects are typically required to consent voluntarily to their own participation in research. Before doing so they must be properly informed about the nature and purpose of the study, what will be expected of them, the possible risks and burdens to them, and other matters in which they can be expected to have an interest.^1^ Where research subjects are young children it is not possible to obtain valid consent from the subjects themselves,^2^ and thus *parental* consent is normally sought. Few would question the need for parental consent in these circumstances. The demand for it is consistent with many other decisions made on behalf of children in society.

In clinical trials involving interventions on *unborn* children the issue of parental consent is less straightforward. In particular, the uniquely intimate relationship between the pregnant woman and her foetus raises difficult questions about the role and importance of *paternal* consent here. In this paper we consider some of these questions. We occasionally refer to BOOSTB4 (Boost Brittle Bones Before Birth).^3^ This EU-funded project aims to evaluate the prenatal and postnatal risks and clinical benefits of a so-far unproven, but potentially promising, stem cell therapy for individuals suffering from brittle bone disease, or osteogenesis imperfecta. It uses mesenchymal stem cells from aborted foetuses which are capable of differentiating and developing into bone cells. The underlying hypothesis is that the intervention will improve the receiver’s bone quality and growth.^4^ However, although this article is inspired by our work within the BOOSTB4 consortium, the discussion we present is by no means confined to the BOOSTB4 clinical trial. Many of the points we make are intended to apply to prenatal research generally.

Maternal consent to prenatal research has been discussed extensively. Paternal consent has received significantly less attention.^5^ In this article, we seek to redress this imbalance. Examining the father’s position in research conducted on the foetus, we ask whether paternal consent ought to be required. We focus on *ethical* issues, and although the paper could be said to raise the profile of the father in the prenatal consent debate, it is certainly not our aim to propose, or call for, changes in the current ethical and legal framework. Our purpose is to call attention to a problem which has been neglected, in our view, by ethicists and policy makers. The paper exploratory in character and provides a starting point for future discussions.

Three further points need to be made about the scope of the discussion. First, by and large we do not explicitly consider the moral and legal status of the *fetus*—a topic on which there already exists a vast literature. Questions about the status of human foetuses are of course highly relevant to the role and limits of paternal consent to prenatal research. Here, however, we confine ourselves to other, as we see it, under-investigated, issues. We are especially interested in differences and similarities in the roles of the father and the mother. We take this to be the natural starting point for a normative discussion concerning the role of paternal consent, but we do not claim that this settles all relevant ethical, regulatory and societal issues. Second, the transfer of stem cells from an aborted foetus to a viable one clearly raises issues about the rights and interests of the *donor* family as well as the mother and father of the recipient foetus. However, we will not address these issues. Instead, we wish to concentrate on the consent of the father of the foetus being intervened upon during any such transfer. Third, since the role of fathers is probably conceived differently in different cultures, it would be interesting to explore if the differences lead to different views about the idea that paternal consent ought to be required in prenatal research. However, empirical investigation of this sort falls outside the normative scope of the present article.

## Conceptual clarifications

It is important to keep track of the different concepts deployed in discussions of prenatal research. These discussions may refer to fathers and paternity. What does ‘paternal’ mean in this context? This issue is more complex than it might at first appear. First of all, the person referred to can be the *biological* father, in the sense that it was his sperm that fertilised the egg which in turn developed into the fetus on which a medical intervention is being contemplated. It is worth noting that a man described in this this way may not (yet) be regarded as a ‘father’ in a narrower sense of that word. In this narrower sense, men *become* fathers when their children are born. The terminological niceties are significant, as the term ‘father’ has rather different connotations from ‘the man responsible for fertilising the ovum’—an expression that would, for example, apply to an anonymous sperm donor.

It is also possible to be a father in the *legal* sense. This may or may not coincide with biological fatherhood. Legal fathers have certain rights and obligations. Although in law the foetus is protected, most obviously by abortion legislation, it may not yet have direct legal standing. But it will eventually develop into an individual that has such a standing. Hence, the legal dimension can become relevant in various kinds of prenatal research projects.

In a third sense of the term, a ‘father’ is someone who stands in a special *social* relation to the child. A ‘social father’ can be deeply involved in the child’s life, and he might care about the interests and welfare of the child just as much as the biological and/or legal father (or indeed mother) does. In this sense the social father is a stakeholder in his own right. What is more, he can offer both practical and emotional support to the pregnant woman.

In practice, these roles often coincide: one and the same person is the biological, legal and social father. But not always. And of course the man recognised as the social or legal father of a child may change during pregnancy and after. Altogether, these complexities of fatherhood mean that it is important, in discussing paternal consent to prenatal interventions, to guard against views that are based on skewed assumptions about ‘the role’ of the father. Potentially, a number of paternal roles need to be distinguished.

As the discussion of the ethics of surrogacy has shown, it is also possible to distinguish between a number of different concepts of motherhood. The woman whose egg is fertilised need not be the person who carries the foetus to term and gives birth to the child, and the woman who gives birth to the child need not be the social mother. Again, in practice these roles are very often combined in one and the same individual. Since paternal consent is the topic here, we will not explore these distinctions further in the present paper.

Same-sex parenting adds another dimension to the issues. The legal and social parents of a child may both be women. Both may be men, as in the case of Elton John and his partner who became fathers with the help of a surrogate mother in California.^6^ Given this, we believe that much of what we say in this paper could be restated in terms of *partner* consent. However, we shall stick with the established, albeit somewhat problematic, way of putting the issue, and talk about paternal consent. (We comment on this way of framing the problem in our final remarks.)

Another important distinction is that between interventions in medical treatment and interventions in clinical trials. The two types of intervention must not be confused, especially in the kinds of case we wish to discuss. The very purpose of a scientific trial evaluating the safety and efficacy of an intervention is (in somewhat simplified terms) to determine whether it is beneficial to the patient. This said, it is difficult to imagine the pregnant woman (or the father) consenting to participation in the kind of research under discussion if they do not think there is a fair chance that this will benefit their foetus/child. This distinction is important because where an intervention has already been proven to be beneficial to the foetus, the father’s position is much weaker, as his veto can only undermine the interests of the child. In the following, however, we will not consider this type of situation.

It is also important to distinguish between aims and effects. In this article we only address interventions undertaken with the aim of *directly benefiting* the foetus. Such an aim will not guarantee that there are no risks and burdens to the foetus, mother or future child. Many interventions do not serve to benefit the foetus at all, and some are associated with risks to the health of the foetus or mother. Here the infamous Thalidomide affair, which highlights risks of adverse effects on pregnant women and their foetuses, needs to be mentioned. Thalidomide was used, among other things, to combat nausea and alleviate morning sickness in pregnant women. The side-effects were devastating, as around 10,000 children were subsequently born with severe disabilities (Kim and Scialli 2011). The episode is a reminder that risks of adverse effects on the foetus should never be neglected, irrespective of an intervention’s aim. Today, however, there is a risk, perhaps, of *overprotecting* pregnant women in research—something which may hamper scientific progress, and which suggests we should not rely only on women to protect their own interests (Denny and Grady 2008).

Why, then, does the researcher’s aim, or intention, matter? Where an intervention only aims to benefit the foetus, it certainly seems reasonable to involve the father in any decision to proceed. By contrast, where the direct aim is to promote the health and wellbeing of the mother, it will be deeply problematic to require paternal consent—something that will be commented on below.^7^

In any discussion of a father’s ‘right’ to be consulted over prenatal interventions, it is crucial to separate the legal and ethical aspects of paternal consent. Here we will focus on the ethical issues, and in particular the values, and interests, and conflicts between them, that need to be considered *before* changes in the legal frameworks are proposed. Eventually, of course, the ethical and legal analyses of paternal consent will need to be brought together, but that is beyond the scope of the present paper.

## Maternal and paternal consent—the rationale

There is no consensus today over the question whether paternal consent ought to be required in prenatal research. Occasionally it is made explicit that there is no such requirement. The Council for International Organizations of Medical Sciences (CIOMS) guidelines, for example, state that the pregnant woman may make decisions “consulting with the father of the fetus, if she wishes” (CIOMS/WHO 2016). The implication is that it is up to the pregnant woman herself to decide whether or not the father should be involved in the decision-making process. Often fathers are not mentioned at all, but there are exceptions to this. The most notable and influential exception is the US Common Rule, which states: “If the research holds out the prospect of direct benefit solely to the fetus” maternal *and* paternal consent is required. The father need not consent, however, “if he is unable to consent because of unavailability, incompetence, or temporary incapacity or the pregnancy resulted from rape or incest” (§46.204 (e)).

For the pregnant woman (henceforth ‘the mother’) things are very different. There is broad consensus that research into prenatal therapy requires her informed consent.^8^ What rationale underlies this requirement? Arguably, it revolves in part around the pregnant woman’s role as a stakeholder, but several other interests are at stake. Below, a list of interests which, individually or in combination, could motivate the requirement of maternal informed consent is presented. Some are perhaps more convincing than others. The list helps us to sort out key rights and interests—rights and interests that can then be compared with those, potentially, of the father.

1. The pregnant women’s bodily integrity is clearly at risk. Infusing a drug to the foetus involves an intrusion to *her* body as well. If such a procedure were to be conducted without her approval it would constitute a serious violation of her right to bodily integrity. Actually, this is perhaps the most paradigmatic example of the conditions under which informed consent is required.^9^
2. The intervention can also harm the mother. Following complications, she could, for example, suffer from an infection or some other adverse health effect. In theory, the stem cell therapy might result in her developing cancer. Depending on the kind of intervention studied, one can picture a number of risks.
3. The procedure as such may involve some degree of pain and/or discomfort to the pregnant woman. That might add a *burden*. In addition, tests, monitoring and follow-up examinations might be needed. These can be very time-consuming and burdensome. In some trials, tests might be required around the clock for days. Other trials may require more frequent visits to the hospital than would otherwise be needed. Although these measures are typically regarded as safety measures—undertaken for her and the foetus’s sake—one should not downplay their potential impact on the woman.
4. Consultation with the mother can also be seen as a way of respecting her as an autonomous person, with a special interest in, and relation to, the health and wellbeing of her foetus/child. A stronger way of putting it would be to say that it is *her* decision to make. Hence, denying her the opportunity to decide would, according to this line of reasoning, constitute a violation of her autonomy and be disrespectful.^10^
5. The procedure (whether it involves surgery, a drug, or an infusion) puts the foetus at risk. Besides well-documented risks of miscarriage, the foetus may suffer from yet unknown adverse effects. As already mentioned, we will not address the interests of the foetus as such. Its wellbeing, however, will be of concern to the mother. In itself, one person’s merely caring about what happens to another provides no moral or legal grounds for requiring consent from that person. In this case, however, the consent is closely related to the kinds of decision parents are expected to make for their children.
6. The outcome of the trial—for both the foetus and the future child—is likely to affect the mother’s future quality of life. If, for example, BOOSTB4 is a success, the mother’s life is likely to become much easier than it would have been had she never participated in the trial. Conversely, if things do not go as intended, the mother’s life might actually turn out worse than it would have been if she had not participated.^11^
7. When the intervention targets a genetic disorder, a genetic analysis will be required to determine whether the subject is suitable for inclusion in the trial. This analysis will reveal a large amount of personal information about the mother. Other kinds of information can also be collected that threaten the mother’s privacy.^12^
8. Once the child is born, the woman will normally become its legal representative. There are exceptions, of course, but these are very rare, especially in the situations we are examining here. This means that generally the woman will acquire both rights and obligations in relation to the child. In this sense, she has an additional interest in what happens to the foetus.

Which of these eight considerations are relevant to the issue of paternal consent? The first three are essentially linked to effects on the mother. There is no straightforward sense in which they apply to the father. The relevance of consideration (4) to the father can be debated, and we shall leave it on the side for now. But considerations (5)–(8) all seem to apply to the father as well as to the mother. Indeed, there is no obvious reason to suppose that they would be less relevant to the father than the mother. The kind of paternity involved is important: where a father is the biological, social and legal father of the foetus/child, all four considerations clearly gain traction.

We are not suggesting, of course, that the sheer number of considerations justifies a requirement for consent (also) from the father. Nor are we assuming that (5)–(8) are as important, in their own way, as (1)–(3). Our claim is merely that the last four considerations need to be considered: each presents a *prima facie* reason for requiring paternal consent, and they therefore need to be addressed, and shown to be inapplicable, in cases where it is decided that the father’s consent is unnecessary.

Other factors that could motivate a paternal consent requirement do not concern the fathers’ rights and interests, but are linked with the feasibility of the study. What are these ‘additional considerations’, as we prefer to call them? In some cases it may not be possible to complete a trial if the father is reluctant, or does not want, his child to participate. This is most obvious in trials that continue after the birth of the child. Many prenatal interventions require follow-up assessments long after the child is born. This might introduce a right for the father to veto participation; and if it is known beforehand that the father will veto continued participation of his child after the birth, it will make little sense to enrol the woman/foetus. Potentially, the question of enrolment could damage the parents’ relationship, or create tension between the parents and health care professionals, and of course this may have a negative impact on the child. However, it remains an open question what will benefit the child most, given the possibility that an intervention carried out, albeit against the father’s wishes, might turn out to be successful.

Without attempting further to assess the relative weights of considerations (1)–(8), and the strength of the additional considerations mentioned above, we shall now ask whether they can serve as grounds justifying a demand for paternal consent.

## The case for paternal consent—does it make sense?

There is clearly a partial overlap between considerations that justify the maternal consent requirement and those justifying a requirement for paternal consent. Few would deny that the maternal case is significantly stronger than the paternal case, but this does not settle the question whether consent should also be required from the father. In fact, given the considerations sketched above, the burden of proof arguably remains on those who deny the father the opportunity to provide or withhold his consent.

There is a normatively relevant asymmetry between the pregnant woman and the father. While it is unproblematic to permit a woman to veto participation in a trial out of concern either for herself or for her foetus and child, it is very controversial to hold that the father ought to be allowed a veto purely for the sake of the pregnant woman. This would be paternalistic, in that it mandates one person to set limits for another person for that second person’s sake even though the first person is not a legal representative of the second.^13^ In practice, of course, it can be very difficult to tell why a person has vetoed a decision. Established standards for informed consent do not require consenters to explain why they have declined to participate, and this goes for legal representatives as well. In fact, such a requirement would conflict with the voluntary requirements found in influential codes of research.

Let us assume to begin with that we are dealing with a ‘father’ in all three senses of the term—biological, social and legal. This after all is the background against which the strongest case can be made for paternal consent. What reasons (if any) are there to deny such a father the right to consent to, or withhold his consent from, a prenatal research procedure? Simply stating that the decision is *the mother’s decision* to make obviously begs the question, and does not clarify anything with respect to the moral grounds for consent, except the biological mother’s autonomy. At the same time, it is arguably only the fact that there is room for conflicting interests between the father and mother—and the fact that the mother’s interests are stronger—that justifies the view that the mother’s, but not the father’s, consent must be obtained.

If we assume, furthermore, for the sake of argument, that the foetus will develop in a fully artificial womb, the father and the mother would arguably have equally strong claims to be consulted—at least, if we deny that there are interests otherwise linked to being a mother or father. In this context it seems reasonable to *require* consent from both parents: considerations (1)–(3) no longer apply. What this indicates is that the interests at stake are sufficient to require informed consent, and that only a competing and stronger maternal interest might justify denying the father the right to be asked for his consent.

What would that stronger maternal interest be? Here it is important to keep in mind that we are *not* discussing interventions aiming ‘merely’ to benefit the pregnant woman directly. In such cases paternal consent would be problematic, as it would limit the woman’s autonomy and her access to, say, experimental treatment for a severe condition from which she suffers.

Does a requirement for paternal consent infringe the mother’s autonomy in other ways? It may do so indirectly via its effect on the mother’s decision as to whether to have an abortion. Where we are dealing with severe genetic diseases, for example, the mother might wish to continue the pregnancy only if there is a chance of avoiding, or mitigating the seriousness of, the disease by participating in the trial. A requirement for paternal consent would then imply that the father can indirectly be given a decisive role in the mother’s decision to choose to terminate the pregnancy.

This particular problem dissolves if a *principle of separation* is observed. This principle says that the woman’s decision as to whether or not she will participate in the prenatal research must be made *after* she has decided to continue with the pregnancy.^14^ This solution may look attractive, but it introduces problems. First, it may not be possible to withhold information about an upcoming trial. Via the Internet, and with the help of patient advocate groups and other interested parties, such information may be spread widely and rapidly. Secondly, one might question whether it is ethical to withhold such information from the expectant mother, since that will arguably reduce the alternatives open to her or limit her opportunities to choose one or some of them. In other words, in ideal circumstances the father’s veto will not influence the mother’s decision about abortion, but society still imposes a constraint by enforcing the principle of separation.

If the case for requiring paternal consent is convincing, the question immediately emerges whether this requirement should be imposed only where it can be demonstrably supported, in the case in question, with reference either to considerations (1)–(8) or to the additional conditions spelled out earlier. The argument may be, for example, that if the genetic analysis alone provides grounds for paternal (and maternal) consent, one should require informed consent from the biological father. If, at the same time, conditions associated with the social or legal role are met, multiple paternal consents from different persons may be necessary. Applying the same distinctions to the mother, it is true that we could, potentially, end up with six different people consenting in a case of prenatal research. This is not only unrealistic, of course, but hardly something we would have reason to require, should that situation ever occur. The unusual scenario envisaged nonetheless illustrates that the relation between being a stakeholder and being permitted to give or withhold consent is anything but straightforward.

There is no safe middle way. On the one hand, we are at risk of infringing the woman’s right to decide whether or not to participate in research. After all, by demanding paternal consent we give the father an opportunity to veto that participation.^15^ On the other hand, a policy of not requiring the father’s informed consent comes at the price of not respecting his rights or interests.^16^

## Concluding remarks

Once more, it needs to be stressed that we are not proposing a new policy, nor any changes in current regulations. We want to raise and explore an ethical issue which has, in our view, been neglected by policy makers and legislators. Intuitively, there is at least *a* case to be made for requiring paternal consent in prenatal research. That case, roughly speaking, revolves around privacy issues, the father’s future legal responsibilities, and the likelihood that he will care about the health and wellbeing of his future child. These factors in the decision problem should all be recognised, as should the fact that they can in principle be trumped by other considerations. These conditions, however, need to be made explicit, explored and evaluated.

We believe that the issue of paternal consent to prenatal research, which call for more research and debate, is too fundamental to be handled by ethical review boards alone. The deliberations of ethical review boards need to be sensitive to the eight considerations we have discussed. It is also important to anticipate and examine pragmatic issues, as there are cases in which the success of the trial is conditional on the father’s consent to the trial’s continuation after the child is born. Should such a pragmatic element be built into ethical codes? Could it be, without violating the autonomy of the pregnant woman?

As we have noted, we have framed the issues addressed in this article in terms of paternal consent rather than partner consent. We realise that for various reasons—most obviously, conception within same-sex relationships—this is an artificial restriction, and we would therefore like to make it clear that, in our view, future codes will need to be drafted in terms giving due respect to partner involvement and partner consent.^17^

## Footnotes

1 See, for example, the conditions set out in Article 13 of the Additional Protocol to the Convention on Human Rights and Biomedicine, concerning Biomedical Research (CETS 195).

2 Depending on age and level of maturity, the child can be given a right to veto participation. When the participant is adolescent his or her consent is occasionally required. Also, ‘assent’ (informed permission) can be required: see (AAP Committee on Bioethics 1995).

3 See https://www.boostb4.eu. The BOOSTB4 trial raises ethical issues beyond those considered in this paper: see (Götherström et al. 2017).

4 The BOOSTB4 proposal states that the primary outcome of the trial is safety, and that secondary outcomes relate to efficacy (fracture frequency, growth, bone mineral density, clinical status of OI and biochemical bone turnover).

5 We have conducted literature searches on ‘paternal consent’ and ‘parental consent’ on PubMed and Google Scholar. The published studies identified differed in many ways from this paper, which focuses on the conditions under which the informed consent of the biological, legal and social father should be required in prenatal research on unproven but promising stem cell therapies. A recent, but rare, exception is a general study of women's views on a paternal consent requirement for biomedical research in pregnancy (Sullivan et al. 2018).

6 https://www.bbc.co.uk/news/entertainment-arts-12084650 (accessed 26 November, 2018).

7 Here it is assumed that the woman is autonomous, and hence is capable to decide. Had this not been the case, due to an accident or illness for example, the father might (depending on circumstances) be allowed or even required to act as a surrogate decision maker. In those cases, he is expected to protect the interests of the woman. We are here not commenting that kind of situation.

8 In strictly clinical contexts, there are cases where pregnant women have been subjected to coercive measures (Mathieu 1991). This is very problematic to say the least, but it is a problem that falls outside of the scope of this article.

9 According to the current Swedish Ethical Review Act (2003:460), for example, this follows.

10 Even so, it must be noted that allowing her to pursue her autonomous interests might in some situations threaten the interests of the foetus; where abortion is being contemplated, there is typically a conflict of interest between the foetus and the pregnant woman.

11 Notice that we are not interested here in the foetus as a stakeholder—not because this is not interesting or important, but simply because if falls outside the scope of the present article.

12 Obviously, this point applies to fathers, too. The information may even indicate, for example, that the social or legal father is not the biological father, and this may have significant (and unwanted) consequences.

13 A very much more morally questionable and problematic reason would be to frustrate the wishes of the mother, or even punish her, in response to a real or imagined harm inflicted on him by her.

14 The National Commission for the Protection of Human Subjects of Biomedical and Behavioral Research: Research involving children. 1977.

15 Needless to say, the father has no right to force the mother to participate, and nothing we have said so far implies that this is the case.

16 We have not been able to identify any alternative that is without problems. For example, it may be proposed that ethical nudge by including the possibility of parental consultation (including both parents) in the consent form while not granting veto for the father would have been a possibility and a better solution. But this proposed solution is not taking the father´s moral interests seriously. We are not suggesting that they trump all other considerations, only that they deserve to be discussed; and the aim of this paper is to call attention to this issue by comparing the interests of various categories of fathers and mothers.

17 The discussion of the paper takes as a starting point situations where a pregnant woman has a male partner who is the biological, social and legal father of the child, and no one else is involved in the pregnancy. We recognize that variants of this scenario, involving among other things same-sex relationships, multiple partners and surrogacy, raise further questions about collective consent, and we are grateful to Paul, Liz and Louis Robinson for pointing out how difficult it is to generalise about consent requirements beyond those applying to the biological mother.
